# A call to arms: Mustering secondary metabolites for success and survival of an opportunistic pathogen

**DOI:** 10.1371/journal.ppat.1007606

**Published:** 2019-04-04

**Authors:** Nicholas Raffa, Nancy P. Keller

**Affiliations:** 1 Department of Medical Microbiology and Immunology, University of Wisconsin-Madison, Madison, Wisconsin, United States of America; 2 Department of Bacteriology, University of Wisconsin-Madison, Madison, Wisconsin, United States of America; McGill University, CANADA

## Introduction

**Fig 1:**
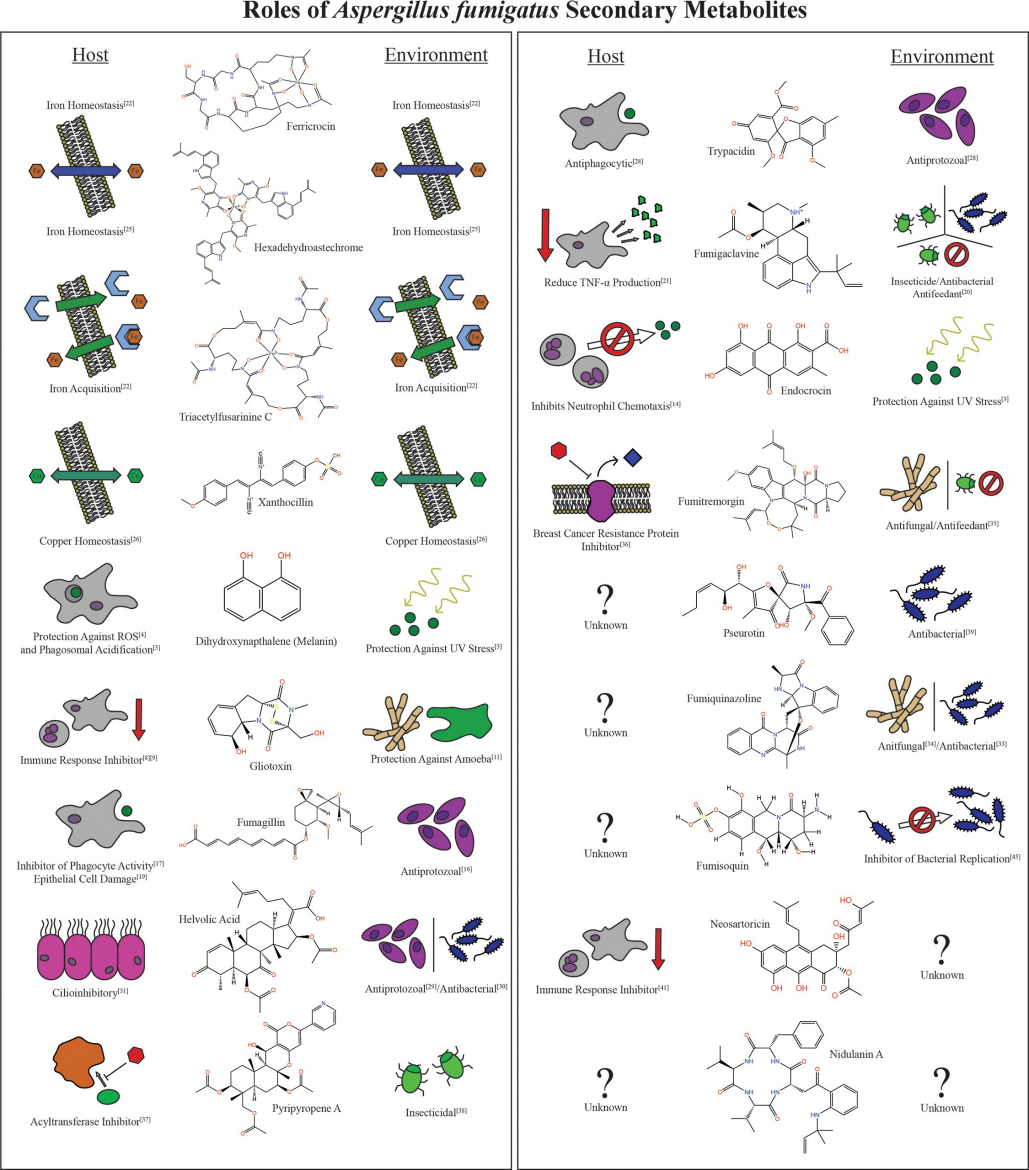
Roles of *Aspergillus fumigatus* secondary metabolites. A list of the secondary metabolites produced by *A*. *fumigatus*, flanked by their proposed roles in the environment (right) and the host (left). Metabolites with a “?” indicate that the compound has not been examined in a niche. Bracketed numerals (e.g., [22]) indicate the reference associated with the role of the metabolite. Nidulanin A is a proposed metabolite produced by *A*. *fumigatus*, whereas all other metabolites are characterized end-product metabolites from a biosynthetic gene cluster. ROS, reactive oxygen species; TNF-α, tumor necrosis factor alpha.

*Aspergillus fumigatus* is a ubiquitous saprophytic mold able to grow on a diversity of material ranging from decayed organic matter in the environment to space station cupolas [1]. Yet this fungus is equally adept as a serious opportunistic pathogen, causing pulmonary aspergillosis and the more deadly invasive aspergillosis (IA). There are an estimated 3,000,000 cases of pulmonary aspergillosis annually and more than 200,000 cases of IA each year reaching a mortality rate of up to 90% in the most susceptible populations [2]. Difficulties in treating IA include delayed detection and increasing resistance to antifungal treatment. Like many opportunistic fungi, there is no one gene that makes *A*. *fumigatus* such a threatening pathogen. One unique feature of this pathogen is its arsenal of small molecules that impact disease development. Secondary metabolites are characterized as bioactive molecules of low molecular weight that are not required for growth of the organism but instead aid survival in harsh environments, resisting desiccation and UV stress and improving competition with other microbes. For *A*. *fumigatus*, these benefits extend to aiding growth not only in the environment but in the human body as well. Some secondary metabolites combat the host immune system by affecting immune cell function or by shielding the fungus against host attack, whereas others allow the fungus to acquire essential, scarce cofactors. The following synopsis of secondary metabolites produced by the opportunistic human pathogen *A*. *fumigatus* highlights how microbial metabolites, although undoubtedly evolved as environmental protectants, can impact infectious disease development (Fig 1). Although we delineate the roles of each metabolite by category for ease of discussion (e.g., “on the offensive,” “scavenging the battlefield,” “arms race”), the reader should note that each metabolite may have several biological roles for the fungus, in part illustrated in Fig 1.

## On the offensive: How *A*. *fumigatus* combats the immune system

Once inside the host, *A*. *fumigatus* must survive interactions with components of the immune system by avoiding, suppressing, or weakening the immune response. The following secondary metabolites have been shown to impact disease or interactions with the immune system through such mechanisms.

### Dihydroxynapthalene melanin

Dihydroxynapthalene (DHN) melanin is a polymer consisting of 1,8-dihydroxynapthalene, found on the conidial surface. As an environmental benefit, DHN melanin helps to prevent desiccation of spores and confers resistance to UV radiation [3]. In the host, DHN melanin protects the conidia by scavenging reactive oxygen species [4], reducing phagosomal acidification in alveolar macrophages [5], and inhibiting apoptosis in epithelial cells [6]. When the polyketide synthase gene (*pksP*/*alb1*) responsible for the initial step of melanin production is deleted, there is a loss of spore pigment, a defect in virulence in intravenously injected immunocompetent murine models, and rapid killing spores in macrophage models [4]. Recently, DHN melanin has been described as a pathogen-associated molecular pattern, in that a C-type lectin receptor expressed in myeloid cells and CD31+ endothelial cells in humans recognizes DHN melanin and has been shown to have a protective role against disseminated infection in immunocompetent mice and recipients of stem cell transplants [7].

### Gliotoxin

Gliotoxin is an epidithiodioxopiperazine that has been extensively studied in the context of infection. Gliotoxin inhibits activity of proteins that contain susceptible free thiols such as the host NADPH oxidase, a protein complex necessary for the generation of antimicrobial reactive oxygen species [8]. Gliotoxin has also been shown to inhibit nuclear factor-kappa B (NF-κB)-mediated transcription of cytokine genes and decrease cytotoxic activities of T lymphocytes [9]. *A*. *fumigatus* is resistant to its own toxin through a protective enzyme encoded in the gliotoxin cluster [10]. More recently, this metabolite has been shown to suppress the macrophage immune response by preventing integrin activation, interfering with actin dynamics, and impairing phagocytosis through affecting phosphoinositide metabolism [11]. When the gliotoxin nonribosomal peptide synthetase gene, *gliP*, is deleted, there is an attenuation of virulence in non-neutropenic murine models of IA but not in neutropenic murine models [12].

### Endocrocin

Endocrocin is a polyketide that is localized to the conidia during growth [13]. Using an in vivo zebrafish assay, endocrocin was found to directly affect immune cells by inhibiting neutrophil chemotaxis [14]. When the polyketide synthase gene *encA* is deleted, there is an attenuation of virulence using the *Drosophila melanogaster* IA model [15]. Endocrocin belongs to a common class of anthraquinones and is closely related to emodin, a precursor in the trypacidin pathway that has been associated with mediating neutrophil apoptosis [15]. Although an exact role for endocrocin has not been established in nature, several related metabolites provide UV protection to fungi, similar to the role of DHN melanin [3].

### Fumagillin

Fumagillin is a monoterpenoid, amoebicidal toxin with valuable pharmaceutical potential due to its inhibitory activity against methionine aminopeptidase-2, making it useful for the treatment of microsporidiosis [16]. The toxin has been found to suppress the immune response of *Galleria mellonella* by inhibiting the activity of phagocytes [17] and reduces the ability of the insect immune cells to kill opsonized *Candida albicans* cells and phagocytose *A*. *fumigatus* conidia [17]. In addition, fumagillin also reduces the ability of hemocytes to take up oxygen and inhibits the translocation of p47 protein [17], an essential component of the NADPH oxidase complex. Fumagillin administered to insect larvae increases the susceptibility of the larvae to *A*. *fumigatus* [18]. Recently, virulence assays with an *A*. *fumigatus* fumagillin deletion mutant strongly support a role for this toxin in epithelial cell damage during IA [19].

### Fumigaclavines

Fumigaclavines are ergot alkaloids, a class of compounds known to act as feeding deterrents and exhibit insecticidal and bactericidal activities [20]. Using the *G*. *mellonella* insect model for IA, it was found that a strain of *A*. *fumigatus* deficient in all ergot alkaloid production, Δ*dmaW*, resulted in a significantly reduced virulence. Strains that were still able to produce ergot alkaloids, but not fumigaclavine C, were significantly less virulent than wild type but still more virulent than the strain in which there was no production of ergot alkaloids, suggesting a role of the end product fumigaclavine C in virulence [20]. Fumigaclavine C has also been shown to inhibit the production of the pro-inflammatory cytokine tumor necrosis factor alpha (TNFα), suggesting a mechanism of action for the molecule [21].

## Scavenging the battlefield: How *A*. *fumigatus* acquires essential micronutrients

Secondary metabolites regulate key aspects of micronutrient homeostasis and allow *A*. *fumigatus* to continue normal cellular function by meeting the needs for the trace elements such as copper and iron. Both are toxic in high doses but are necessary for essential cellular processes such as respiration and branched-chain amino acid biosynthesis. The ability to acquire these micronutrients is directly related to the ability of *A*. *fumigatus* to cause disease.

### Siderophores

Siderophores produced by *A*. *fumigatus* are characterized by their hydrodroxamate moieties and function in high-affinity iron uptake and storage mechanisms. Extracellular siderophores fusarinine C and triacetlyfusarinine C are secreted into the environment, where they bind Fe^3+^ and transport it back into the cell. Intracellular siderophores ferricrocin and hydroxyferricrocin are responsible for iron storage and homeostasis. When the enzyme responsible for the first step in siderophore biosynthesis *sidA* is deleted, both extracellular and intracellular siderophore production is abolished. The *sidA* deletion grows poorly under iron-limiting conditions [22] and displays increased sensitivity to hydrogen peroxide. In addition, this mutant was found to be highly attenuated in virulence using a neutropenic murine model [23], suggesting that proper iron acquisition is essential for disease progression in the host.

### Hexadehydroastechrome

Hexadehydroastechrome (HAS) is a tryptophan-derived secondary metabolite that binds to iron. Overexpressing the transcription factor present within the HAS biosynthetic gene cluster results in an increase in both siderophore and HAS production in addition to increased virulence in a neutropenic murine model [24]. HAS regulates fungal iron homeostasis circuitry, aligning iron acquisition and consumption pathways with secondary metabolite expression [25], including the newly discovered xanthocillin gene cluster [26].

### Xanthocillins

Xanthocillins are tyrosine-derived metabolites that contain a characteristic isocyanide functional group and have been recently shown to be produced by the *xan* cluster in *A*. *fumigatus*. Overexpression of the transcription factor present within the cluster results in an increased production of isocyanides and a defect in copper-dependent pigmentation indicating a possible link of this cluster to copper homeostasis [26]. The isocyanides produced by *A*. *fumigatus* may represent a unique mechanism, on top of the canonical copper regulatory system [27], to maintaining copper homeostasis for this pathogen.

## Arms race: How *A*. *fumigatus* uses secondary metabolites to compete in the environment and host

Several secondary metabolites have no known effect or have not been tested for effects on virulence or interactions with the immune system but have only been shown to provide an advantage to *A*. *fumigatus* when competing with other microbes in the environment.

### Trypacidin

Trypacidin is an anthraquinone that has been found to have antiprotozoal, cytotoxic, and antiphagocytic properties. The compound displays activity against *Toxoplasma gondii* and *Trypanosoma cruzi* in vitro that causes toxoplasmosis and Chagas disease, respectively. Deleting the polyketide synthase essential for trypacidin production eliminates production of the metabolite and coincides with an increase in phagocytosis when challenged with *Dictyostelium discoideum* and macrophages, indicating that trypacidin acts as an antiphagocytic metabolite [28]. The trypacidin pathway shows redundant synthesis to the endocrocin pathway, where both contribute to final endocrocin synthesis in some strains of *A*. *fumigatus* [15].

### Helvolic acid

Helvolic acid is a fusidane antibiotic that exhibits in vitro antiprotozoal activity against the trypanosome *Trypanosoma brucei brucei* GUTat3.1, the causative agent of African sleeping sickness [29], and helvolic acid derivatives exhibit antibacterial activity against *Streptococcus agalactiae* and *Staphylococcus aureus* [30]. In addition, helvolic acid also affects mammalian cell lines, decreasing the beat frequency of ciliated respiratory epithelium, a process important in preventing colonization by *A*. *fumigatus* [31].

### Fumiquinazolines

Fumiquinazolines are tryptophan-derived peptidyl alkaloids that have a broad range of activity and accumulate in *A*. *fumigatus* conidia [32]. Fumiquinazoline F isolated from cultures of *Penicillium coryphilum* exhibited activity against *S*. *aureus* and *Micrococcus luteus* [33]. Fumiquinazolines also exhibit antifungal activity with fumiquinazoline H and I isolated from *Acremonium sp*. showing weak antifungal activity against *C*. *albicans* [34].

### Fumitremorgins

Fumitremorgins belong to the diketopiperazine alkaloids class of compounds and contain a unique, 8-membered endoperoxide ring. Fumitremorgin B has been found to have in vitro antifungal activity against a variety of phytopathogenic of fungi [35]. In addition, fumitremorgin B was found to be lethal to brine shrimp and displayed antifeedant activity towards armyworm larvae [35]. Fumitremorgins have also been shown to affect mammalian cells. Fumitremorgin C displays inhibitory activity towards the breast cancer resistance protein, an ATP-binding cassette transporter that is implicated in cellular resistance to anticancer drugs [36].

### Pyripyropene A

Pyripyropene A was discovered during an investigation into inhibitors of acyl-coenzyme A (CoA):cholesterol acyltransferase, a mechanism by which to treat hypercholesterolemia and atherosclerosis [37]. Pyripyropenes were further shown to exhibit in vivo aphicidal activity against the green peach aphid (*Myzus persicae*) during a screen of compounds that act as insecticides [38]. How these activities may relate to aspergillosis has not been assessed.

### Pseurotin

Pseurotin has been shown to be have several antimicrobial and cytotoxic properties. It has been demonstrated to have antibacterial properties when screened against both gram-positive and gram-negative organisms [39]. This metabolite is encoded by an intertwined biosynthetic gene cluster with fumagillin [40] but, unlike fumagillin, was not implicated in epithelial tissue damage [19].

### Neosartoricin

Neosartoricin is a prenylated anthracenone and was discovered following activation of the gene cluster from *A*. *fumigatus* and *Neosartorya fischeri* [41]. The compound was found to have T-cell antiproliferative activity suggesting that the compound functions as an immunosuppressive [41]. Like several metabolites synthesized by *A*. *fumigatus*, the biosynthetic gene cluster is conserved in several pathogenic fungi [42].

### Fumisoquin

Fumisoquin is an isoquinolone alkaloid with biosynthetic machinery that bears a striking similarity to plant berberine bridge enzyme and tetrahydrocannabinol biosynthesis [43]. Deletion of the fumisoquin transcription factor did not impact virulence in a murine infection model [44]. A related isoquinalone metabolite produced by *Aspergillus flavus* stimulates *Aspergillus* species spore germination while inhibiting bacterial growth [45], possibly hinting at a function for fumisoquin.

### Nidulanin A

Nidulanin A is a tetracyclopeptide/isoprene isolated from *Aspergillus nidulans* [46]. The nidulanin A gene cluster is conserved in all *Aspergillus* spp., including *A*. *fumigatus*, although it has not been detected in this fungus [42]. At present, nidulanin A has yet to be tested for any antimicrobial or virulence-related properties.

## Prospective

*A*. *fumigatus* produces a wide variety of small molecules, many of which are demonstrated to impact virulence, others of which have not been investigated, and likely still some of which have yet to be discovered. These molecules are the weapons that *A*. *fumigatus* uses to do battle with the immune system, facilitate the acquisition of essential micronutrients in their environment, and compete with other microbes. It is important to note, however, that *A*. *fumigatus* isn’t alone in producing secondary metabolites that affect virulence. Many of these secondary metabolites are conserved in other pathogenic fungi [38]. Studying secondary metabolites produced by *A*. *fumigatus* will provide insight into understanding not only the chemical arsenal of *A*. *fumigatus* but the chemical arsenal of other pathogenic fungi as well.

## References

[1] Knox BP, Blachowicz A, Palmer JM, Romsdahl J, Huttenlocher A, Wang CC, Keller NP, Venkateswaran K. Characterization of *Aspergillus fumigatus* isolates from air and surfaces of the international Space Station. mSphere. 2016 10 26;1(5)00227–16. 10.1128/mSphere.00227-16 PMC5082629 27830189

[2] Taccone FS, Van den Abeele AM, Bulpa P, Misset B, Meersseman W, Cardoso T, Paiva JA, Blasco-Navalpotro M, De Laere E, Dimopoulos G, Rello J. Epidemiology of invasive aspergillosis in critically ill patients: clinical presentation, underlying conditions, and outcomes. Crit Care. 2015 12;19(1):7. 25928694 10.1186/s13054-014-0722-7 PMC4344741

[3] Nguyen KH, Chollet-Krugler M, Gouault N, Tomasi S. UV-protectant metabolites from lichens and their symbiotic partners. Nat Prod Rep. 2013;30(12):1490–508. 10.1039/c3np70064j 24170172

[4] Brakhage AA, Liebmann B. *Aspergillus fumigatus* conidial pigment and cAMP signal transduction: significance for virulence. Medical mycology. 2005 1 1;43(1):75–82. 10.1080/13693780400028967 16110796

[5] Jahn B, Langfelder K, Schneider U, Schindel C, Brakhage AA. PKSP‐dependent reduction of phagolysosome fusion and intracellular kill of *Aspergillus fumigatus* conidia by human monocyte‐derived macrophages. Cell microbiol. 2002 12;4(12):793–803. 12464010 10.1046/j.1462-5822.2002.00228.x

[6] Berkova N, Lair-Fulleringer S, Féménia F, Huet D, Wagner MC, Gorna K, Tournier F, Ibrahim-Granet O, Guillot J, Chermette R, Boireau P. *Aspergillus fumigatus* conidia inhibit tumour necrosis factor-or staurosporine-induced apoptosis in epithelial cells. Int Immunol. 2005 12 15;18(1):139–50. 10.1093/intimm/dxh356 16357007

[7] Stappers MH, Clark AE, Aimanianda V, Bidula S, Reid DM, Asamaphan P, Hardison SE, Dambuza IM, Valsecchi I, Kerscher B, Plato A. Recognition of DHN-melanin by a C-type lectin receptor is required for immunity to *Aspergillus*. Nature. 2018 3;555(7696):382 10.1038/nature25974 29489751 PMC5857201

[8] Scharf DH, Brakhage AA, Mukherjee PK. Gliotoxin–bane or boon?. Environ Microbiol. 2016 4;18(4):1096–109. 10.1111/1462-2920.13080 26443473

[9] Yamada A, Kataoka T, Nagai K. The fungal metabolite gliotoxin: immunosuppressive activity on CTL-mediated cytotoxicity. Immunol Lett. 2000 1 10;71(1):27–32. 10709782 10.1016/s0165-2478(99)00155-8

[10] Dolan SK, O’Keeffe G, Jones GW, Doyle S. Resistance is not futile: gliotoxin biosynthesis, functionality and utility. Trends Microbiol. 2015 7 1;23(7):419–28. 10.1016/j.tim.2015.02.005 25766143

[11] Schlam D, Canton J, Carreño M, Kopinski H, Freeman SA, Grinstein S, Fairn GD. Gliotoxin suppresses macrophage immune function by subverting phosphatidylinositol 3, 4, 5-trisphosphate homeostasis. MBio. 2016 5 4;7(2):e02242–15. 10.1128/mBio.02242-15 27048806 PMC4817266

[12] Dagenais TR, Keller NP. Pathogenesis of Aspergillus fumigatus in invasive aspergillosis. Clin Microbiol Rev. 2009 7 1;22(3):447–65. 10.1128/CMR.00055-08 19597008 PMC2708386

[13] Lim FY, Hou Y, Chen Y, Oh JH, Lee I, Bugni TS, Keller NP. Genome-based cluster deletion reveals endocrocin biosynthetic pathway in *Aspergillus fumigatus*. Appl Environ Microbiol. 2012 4 6:AEM-07710. 10.1128/AEM.07710-11 PMC3370519 22492455

[14] Berthier E, Lim FY, Deng Q, Guo CJ, Kontoyiannis DP, Wang CC, Rindy J, Beebe DJ, Huttenlocher A, Keller NP. Low-volume toolbox for the discovery of immunosuppressive fungal secondary metabolites. PLoS Pathog. 2013 4 11;9(4)1003289. 10.1371/journal.ppat.1003289 PMC3623715 23592999

[15] Throckmorton K, Lim FY, Kontoyiannis DP, Zheng W, Keller NP. Redundant synthesis of a conidial polyketide by two distinct secondary metabolite clusters in *Aspergillus fumigatus*. Environ microbiol. 2016 1;18(1):246–59. 10.1111/1462-2920.13007 26242966 PMC4750049

[16] Mendoza Y, Diaz-Cetti S, Ramallo G, Santos E, Porrini M, Invernizzi C. Nosema ceranae Winter Control: Study of the Effectiveness of Different Fumagillin Treatments and Consequences on the Strength of Honey Bee (*Hymenoptera*: *Apidae*) Colonies. J Econ Entomol. 2016 12 26;110(1):1–5. 10.1093/jee/tow228 28025388

[17] Fallon JP, Reeves EP, Kavanagh K. Inhibition of neutrophil function following exposure to the *Aspergillus fumigatus* toxin fumagillin. J Med Microbiol. 2010 6 1;59(6):625–33. 20203215 10.1099/jmm.0.018192-0

[18] Fallon JP, Reeves EP, Kavanagh K. The *Aspergillus fumigatus* toxin fumagillin suppresses the immune response of *Galleria mellonella* larvae by inhibiting the action of haemocytes. Microbiology. 2011 5 1;157(5):1481–8. 21349977 10.1099/mic.0.043786-0

[19] Guruceaga X, Ezpeleta G, Mayayo E, Sueiro-Olivares M, Abad-Diaz-de-Cerio A, Aguirre JM, Liu HG, Wiemann P, Bok JW, Filler SG, Keller NP, Hernando FL, Ramirez-Garcia A, Rementeria A. A possible role for fumagillin in cellular damage during host infection by *Aspergillus fumigatus*. Virulence. 2018 9 24 9(1):1548–1561. 10.1080/21505594.2018.1526528 30251593 PMC6177242

[20] Panaccione DG, Arnold SL. Ergot alkaloids contribute to virulence in an insect model of invasive aspergillosis. Sci Rep. 2017 8 21;7(1):8930 10.1038/s41598-017-09107-2 28827626 PMC5567044

[21] Du RH, Li EG, Cao Y, Song YC, Tan RX. Fumigaclavine C inhibits tumor necrosis factor α production via suppression of toll-like receptor 4 and nuclear factor κB activation in macrophages. Life Sci. 2011 8 15;89(7–8):235–40. 10.1016/j.lfs.2011.06.015 21762706

[22] Hissen AH, Wan AN, Warwas ML, Pinto LJ, Moore MM. The *Aspergillus fumigatus* siderophore biosynthetic gene *sidA*, encoding L-ornithine N5-oxygenase, is required for virulence. Infect Immun. 2005 9 1;73(9):5493–503. 10.1128/IAI.73.9.5493-5503.2005 16113265 PMC1231119

[23] Schrettl M, Bignell E, Kragl C, Joechl C, Rogers T, Arst HN, Haynes K, Haas H. Siderophore biosynthesis but not reductive iron assimilation is essential for *Aspergillus fumigatus* virulence. J Exp Med. 2004 11 1;200(9):1213–9. 10.1084/jem.20041242 15504822 PMC2211866

[24] Yin WB, Baccile JA, Bok JW, Chen Y, Keller NP, Schroeder FC. A nonribosomal peptide synthetase-derived iron (III) complex from the pathogenic fungus *Aspergillus fumigatus*. J Am Chem Soc 2013 2 1;135(6):2064–7. 10.1021/ja311145n 23360537 PMC3590312

[25] Wiemann P, Lechner BE, Baccile JA, Velk TA, Yin WB, Bok JW, Pakala S, Losada L, Nierman WC, Schroeder FC, Haas H. Perturbations in small molecule synthesis uncovers an iron-responsive secondary metabolite network in *Aspergillus fumigatus*. Front Microbiol. 2014 10 24;5:530 10.3389/fmicb.2014.00530 25386169 PMC4208449

[26] Lim FY, Won TH, Raffa N, Baccile JA, Wisecaver J, Rokas A, Schroeder FC, Keller NP. Fungal Isocyanide Synthases and Xanthocillin Biosynthesis in *Aspergillus fumigatus*. mBio. 2018 7 5;9(3):e00785–18. 10.1128/mBio.00785-18 29844112 PMC5974471

[27] Wiemann P, Perevitsky A, Lim FY, Shadkchan Y, Knox BP, Figueora JA, Choera T, Niu M, Steinberger AJ, Wüthrich M, Idol RA. *Aspergillus fumigatus* copper export machinery and reactive oxygen intermediate defense counter host copper-mediated oxidative antimicrobial offense. Cell Rep. 2017 5 2;19(5):1008–21. 10.1016/j.celrep.2017.04.019 28467895 PMC5512462

[28] Mattern DJ, Schoeler H, Weber J, Novohradská S, Kraibooj K, Dahse HM, Hillmann F, Valiante V, Figge MT, Brakhage AA. Identification of the antiphagocytic trypacidin gene cluster in the human-pathogenic fungus *Aspergillus fumigatus*. Appl Microbiol Biotechnol. 2015 12 1;99(23):10151–61. 10.1007/s00253-015-6898-1 26278536

[29] Ganaha M, Yoshii K, Ōtsuki Y, Iguchi M, Okamoto Y, Iseki K, Ban S, Ishiyama A, Hokari R, Iwatsuki M, Otoguro K. In vitro antitrypanosomal activity of the secondary metabolites from the mutant strain IU-3 of the insect pathogenic fungus *Ophiocordyceps coccidiicola* NBRC 100683. Pharm Bull. 2016 7 1;64(7):988–90. 10.1248/cpb.c16-00220 27373660

[30] Kong FD, Huang XL, Ma QY, Xie QY, Wang P, Chen PW, Zhou LM, Yuan JZ, Dai HF, Luo DQ, Zhao YX. Helvolic Acid Derivatives with Antibacterial Activities against *Streptococcus agalactiae* from the Marine-Derived Fungus *Aspergillus fumigatus* HNMF0047. J Nat Prod. 2018 8 2;81(8):1869–76. 10.1021/acs.jnatprod.8b00382 30070829

[31] Amitani R, Taylor G, Elezis EN, Llewellyn-Jones C, Mitchell J, Kuze F, Cole PJ, Wilson R. Purification and characterization of factors produced by Aspergillus fumigatus which affect human ciliated respiratory epithelium. Infect Immun. 1995 9 1;63(9):3266–71. 7543879 10.1128/iai.63.9.3266-3271.1995 PMC173450

[32] Lim FY, Ames B, Walsh CT, Keller NP. Co‐ordination between BrlA regulation and secretion of the oxidoreductase FmqD directs selective accumulation of fumiquinazoline C to conidial tissues in *Aspergillus fumigatus*. Cell Microbiol. 2014 8;16(8):1267–83. 10.1111/cmi.12284 24612080 PMC4114987

[33] Silva MG, Furtado NA, Pupo MT, Fonseca MJ, Said S, da Silva Filho AA, Bastos JK. Antibacterial activity from *Penicillium corylophilum Dierckx*. Microbiol Res. 2004 12 15;159(4):317–22. 15646377 10.1016/j.micres.2004.06.003

[34] Belofsky GN, Anguera M, Jensen PR, Fenical W, Köck M. Oxepinamides A‐C and Fumiquinazolines H‐I: Bioactive Metabolites from a Marine Isolate of a Fungus of the Genus *Acremonium*. Chemistry. 2000 4 17;6(8):1355–60. 10840958 10.1002/(sici)1521-3765(20000417)6:8<1355::aid-chem1355>3.0.co;2-s

[35] Li XJ, Zhang Q, Zhang AL, Gao JM. Metabolites from *Aspergillus fumigatus*, an endophytic fungus associated with *Melia azedarach*, and their antifungal, antifeedant, and toxic activities. J Agric Food Chem. 2012 3 23;60(13):3424–31. 10.1021/jf300146n 22409377

[36] González-Lobato L, Real R, Prieto JG, Álvarez AI, Merino G. Differential inhibition of murine Bcrp1/Abcg2 and human BCRP/ABCG2 by the mycotoxin fumitremorgin C. Eur J Pharmacol. 2010 10 10;644(1–3):41–8. 10.1016/j.ejphar.2010.07.016 20655304

[37] Omura S, Tomoda H, Kim YK, Nishida H. Pyripyropenes, highly potent inhibitors of acyl-CoA: cholesterol acyltransferase produced by *Aspergillus fumigatus*. J Antibiot (Tokyo). 1993 7 25;46(7):1168–9. 8360113 10.7164/antibiotics.46.1168

[38] Goto K, Horikoshi R, Mitomi M, Oyama K, Hirose T, Sunazuka T, Ōmura S. Synthesis and insecticidal efficacy of pyripyropene derivatives focusing on the C-1, C-7, and C-11 positions’ substituent groups. J Antibiot (Tokyo). 2018 5 23:1. 10.1038/s41429-018-0064-9 29789612

[39] Mehedi MA, Molla AH, Khondkar PR, Sultana S, Islam MA, Rashid MA, Chowdhury R. Pseurotin A: an antibacterial secondary metabolite from *Aspergillus fumigatus*. Chem Asian J. 2010 4 1;22:2611–4.

[40] Wiemann P, Guo CJ, Palmer JM, Sekonyela R, Wang CC, Keller NP. Prototype of an intertwined secondary-metabolite supercluster. Proceedings of the National Academy of Sciences. 2013 10 15;110(42):17065–70. 10.1073/pnas.1313258110 PMC3801025 24082142

[41] Yin WB, Chooi YH, Smith AR, Cacho RA, Hu Y, White TC, Tang Y. Discovery of cryptic polyketide metabolites from dermatophytes using heterologous expression in *Aspergillus nidulans*. ACS Synth Biol. 2013 6 11;2(11):629–34. 10.1021/sb400048b 23758576 PMC3795930

[42] Bignell E, Cairns TC, Throckmorton K, Nierman WC, Keller NP. Secondary metabolite arsenal of an opportunistic pathogenic fungus. Phil Trans R Soc B. 2016 12 5;371(1709):20160023 10.1098/rstb.2016.0023 28080993 PMC5095546

[43] Baccile JA, Spraker JE, Le HH, Brandenburger E, Gomez C, Bok JW, Macheleidt J, Brakhage AA, Hoffmeister D, Keller NP, Schroeder FC. Plant-like biosynthesis of isoquinoline alkaloids in *Aspergillus fumigatus*. Nat Chem Biol. 2016 6 1;12(6):419–24. 10.1038/nchembio.2061 27065235 PMC5049701

[44] Macheleidt J, Scherlach K, Neuwirth T, Schmidt‐Heck W, Straßburger M, Spraker J, Baccile JA, Schroeder FC, Keller NP, Hertweck C, Heinekamp T. Transcriptome analysis of cyclic AMP‐dependent protein kinase A–regulated genes reveals the production of the novel natural compound fumipyrrole by *Aspergillus fumigatus*. Mol Microbiol. 2015 4;96(1):148–62. 10.1111/mmi.12926 25582336 PMC4425693

[45] Khalid S, Baccile JA, Spraker JE, Tannous J, Imran M, Schroeder FC, Keller NP. NRPS-derived isoquinolines and lipopetides mediate antagonism between plant pathogenic fungi and bacteria. ACS Chem Biol. 2017 12 18;13(1):171–9. 10.1021/acschembio.7b00731 29182847 PMC5922988

[46] Andersen MR, Nielsen JB, Klitgaard A, Petersen LM, Zachariasen M, Hansen TJ, Blicher LH, Gotfredsen CH, Larsen TO, Nielsen KF, Mortensen UH. Accurate prediction of secondary metabolite gene clusters in filamentous fungi. Proceedings of the National Academy of Sciences. 2013 1 2;110(1):E99–107. 10.1073/pnas.1205532110 PMC3538241 23248299

